# Risk of adverse obstetric outcomes in Japanese women with systemic lupus erythematosus: The Japan Environment and Children’s Study

**DOI:** 10.1371/journal.pone.0233883

**Published:** 2020-05-29

**Authors:** Tsuyoshi Murata, Hyo Kyozuka, Toma Fukuda, Shun Yasuda, Akiko Yamaguchi, Akiko Sato, Yuka Ogata, Masahito Kuse, Mitsuaki Hosoya, Seiji Yasumura, Koichi Hashimoto, Hidekazu Nishigori, Keiya Fujimori, The Japan Environment and Children’s Study Group

**Affiliations:** 1 Fukushima Regional Center for the Japan Environmental and Children’s Study, Fukushima, Japan; 2 Department of Obstetrics and Gynecology, Fukushima Medical University School of Medicine, Fukushima, Japan; 3 Department of Pediatrics, Fukushima Medical University School of Medicine, Fukushima, Japan; 4 Department of Public Health, Fukushima Medical University School of Medicine, Fukushima, Japan; 5 Fukushima Medical Center for Children and Women, Fukushima Medical University School of Medicine, Fukushima, Japan; University of Mississippi Medical Center, UNITED STATES

## Abstract

Systemic lupus erythematosus, simply known as lupus, is associated with adverse obstetric outcomes. This study evaluated the incidence of preterm births (before 37 and 34 weeks), low birthweight infants (<2500 g and <1500 g), small-for-gestational age infants, preterm premature rupture of membranes, and gestational hypertension in mothers with lupus and compared them with those of the Japanese general population. Data from participants in the Japan Environment and Children’s Study who gave birth between 2011 and 2014 were collected. Only participants with singleton pregnancies were included. Adjusted odds ratios for the variables were calculated using a logistic regression model, with a general population as the reference. In total, 88,017 participants were included in the analysis, and 63 of them had lupus. The adjusted odds ratios of preterm births before 37 and 34 weeks, low birthweight infants <2500 g and <1500 g, small-for-gestational age infants, and preterm premature rupture of membranes in the systemic lupus erythematosus group were 8.1 (95% CI, 4.7–14.1), 5.2 (1.6–16.5), 6.5 (3.9–10.8), 5.4 (1.3–22.4), 2.9 (1.4–5.9), and 12.1 (5.7–25.5), respectively. The adjusted odds ratio of gestational hypertension was 1.4 (0.4–4.5). This study revealed increased risk of preterm births, low birthweight infants, small-for-gestational age infants, and preterm premature rupture of membranes in patients with lupus when compared with those in the general population.

## Introduction

Systemic autoimmune rheumatic diseases, including systemic lupus erythematosus (SLE), systemic sclerosis, rheumatoid arthritis, polymyositis, dermatomyositis, and juvenile idiopathic arthritis, commonly affect pregnancy outcomes [1]. As SLE affects women more frequently than men in every age group and ethnic group [2], the prevalence of SLE in women in the UK has been reported to be approximately 50 per 100,000 [2] and the prevalence in women in California was reported as 164 (white), 406 (African American), and 93 (Asian) per 100,000 [3]. The prevalence in women of childbearing age has been reported to be approximately 163 per 100,000 [3]. Women with SLE have a normal fertility rate [4], and one study estimated that approximately 1/1250 of all pregnancies in the United States were to women with SLE [5]. Even though pregnancy outcomes of women with SLE have improved over the years, as the pregnancy loss rate had decreased from 43% to 17% in 40 years by 2000 [5, 6], SLE is still associated with adverse obstetric outcomes, such as preterm births (PTB) and fetal growth restriction (FGR), which possibly lead to low birthweight infants (LBW) and small-for-gestational age infants (SGA) [5, 7–9]. PTB and related LBW are leading causes of neonatal mortality and morbidity, which account for 75% of perinatal mortality and more than half of the long-term morbidity [10–12].

Appropriate interventions for PTB and specialized neonatal care for LBW and SGA are necessary to improve neonatal outcomes. Therefore, the ability to accurately assess the risk of adverse outcomes is highly important for physicians and women with SLE. Several reports have identified risk factors for adverse obstetric outcomes in pregnant women with SLE, such as thrombocytopenia, proteinuria, antiphospholipid syndrome, and anti-Ro/SSA or anti-La/SSB antibodies [9, 13–15]. Although several studies have demonstrated the risk of PTB [1, 5, 7–9, 16], FGR [5, 7, 8], preterm premature rupture of membranes (pPROM) [1, 16] and maternal hypertension and preeclampsia [5, 7–9, 16] in women with SLE, the risk of LBW and SGA have not been clearly studied. Furthermore, few studies have been conducted to clarify the incidence of adverse obstetric outcomes in the Asian population [8], despite the significant difference in prevalence of SLE among different ethnic groups [2, 3, 8].

Therefore, the aim of this study was to evaluate the effects of maternal SLE on the incidence of PTB, LBW, and SGA, and compare the results with those of the general population using data from a nationwide Japanese prospective birth cohort study. In addition, this study aimed to evaluate the effects of maternal SLE on the incidence of pPROM and gestational hypertension (GH), which is thought to be related to the incidence of PTB.

## Materials and methods

Data from women enrolled in the Japan Environment and Children’s Study (JECS), a nationwide and government-funded prospective birth cohort study started in January 2011 to investigate the effects of environmental factors on children’s health [17, 18], was used for the study. The inclusion criteria were: (1) residing in the study area at the time of recruitment and expected to continually reside in Japan for the foreseeable future, (2) an expected delivery date between August 1, 2011, and mid‐2014, and (3) capable of participating in the study without difficulty (i.e., able to comprehend the Japanese language and complete the self-administered questionnaire). In addition, only women with singleton pregnancies were included in the present study.

Two recruitment protocols were applied and included recruitment at the time of the first prenatal examination at cooperating obstetric facilities and recruitment at local government offices through a pregnancy journal, called the Maternal and Child Health Handbook, that is given to all expecting mothers in Japan before they receive municipal services for pregnancy, delivery, and childcare. Written informed consent was obtained from all participating women.

The JECS protocol was reviewed and approved by the Ministry of the Environment’s Institutional Review Board on Epidemiological Studies and by the Ethics Committees of all participating institutions. The JECS was conducted in accordance with the Helsinki Declaration and other nationally valid regulations and guidelines.

### Data collection

The current analysis used the data set released in June 2016 (data set: jecs-ag-20160424). Specifically, we used three types of data: (1) M-T1, obtained from a self-reported questionnaire that was collected during the first trimester (the first questionnaire) and that included questions regarding maternal medical background; (2) M-T2, obtained from a self-reported questionnaire that was collected during the second or third trimester (second questionnaire) and that included partner lifestyle and socioeconomic status; and (3) Dr-0m, collected from medical records provided by each participant’s institution and that included obstetrical outcomes such as gestational age, birthweight, and obstetric complications. Patients with SLE were diagnosed based on the American College of Rheumatology revised criteria for the classification of systemic lupus erythematosus [19], which are widely used in Japan, and they were collected based on medical records.

### Obstetric outcomes and confounding factors

PTB was classified into two categories: before 37 weeks and before 34 weeks. LBW was classified into two categories: LBW < 2500 g and LBW < 1500 g. SGA was defined as a birthweight below −1.5 standard deviations corrected for gestational age and sex according to “New Japanese neonatal anthropometric charts for gestational age at birth” [20]. pPROM was defined as spontaneous rupture of membranes before 37 weeks. The GH was defined as the new onset of elevated blood pressure ≥140/90 mmHg occurring after 20 weeks of pregnancy in an otherwise normotensive woman. The following items were analyzed as potential confounding factors: maternal age, body mass index (BMI) before pregnancy, parity, maternal smoking status, maternal educational status, and annual household income. Participants were divided into three groups (maternal age <20 years, 20–29 years, and ≥30 years). Body mass index before pregnancy was categorized into three groups (<18.5, 18.5–25.0, >25.0 kg/m^2^). Parity was categorized into two groups (nulliparous and multiparous). Participants were requested to provide information about their smoking status by choosing one of the following: “kept smoking during pregnancy,” “never smoked,” “quit smoking before pregnancy,” or “quit smoking during early pregnancy.” The participants who chose “kept smoking during pregnancy” comprised the smoking category, while the other participants comprised the non-smoking category. The educational status of the mother was categorized into four groups based on the number of years of education (junior high school, <10 years; high school, 10–12 years; professional school or university, 13–16 years; and graduate school, ≥17 years). Annual household income was categorized into four levels (<2,000,000, 2,000,000–5,999,999, 6,000,000–9,999,999, and ≥10,000,000 JPY). Confounding factors in this study were chosen based on clinical importance [21–23].

### Statistical analysis

Participant characteristics were summarized according to the presence of SLE. Student’s t-test was used to compare continuous variables between each group, and the chi-square test was used to compare categorical variables. Adjusted odds ratios (aORs) and 95% confidence intervals (CIs) for PTB, LBW, SGA, pPROM, and GH were calculated using a multiple logistic regression model. The odds ratios were adjusted for maternal age, BMI before pregnancy, parity, maternal smoking status, maternal educational status, and annual household income.

SPSS version 26 (IBM Corp., Armonk, NY, USA) was used for the statistical analysis. A p-value of less than 0.05 was considered to indicate statistical significance.

### Ethics approval

The JECS protocol was approved by the Ministry of the Environment’s Institutional Review Board on Epidemiological Studies and by the Ethics Committees of all participating institutions.

## Results and discussion

**Fig 1:**
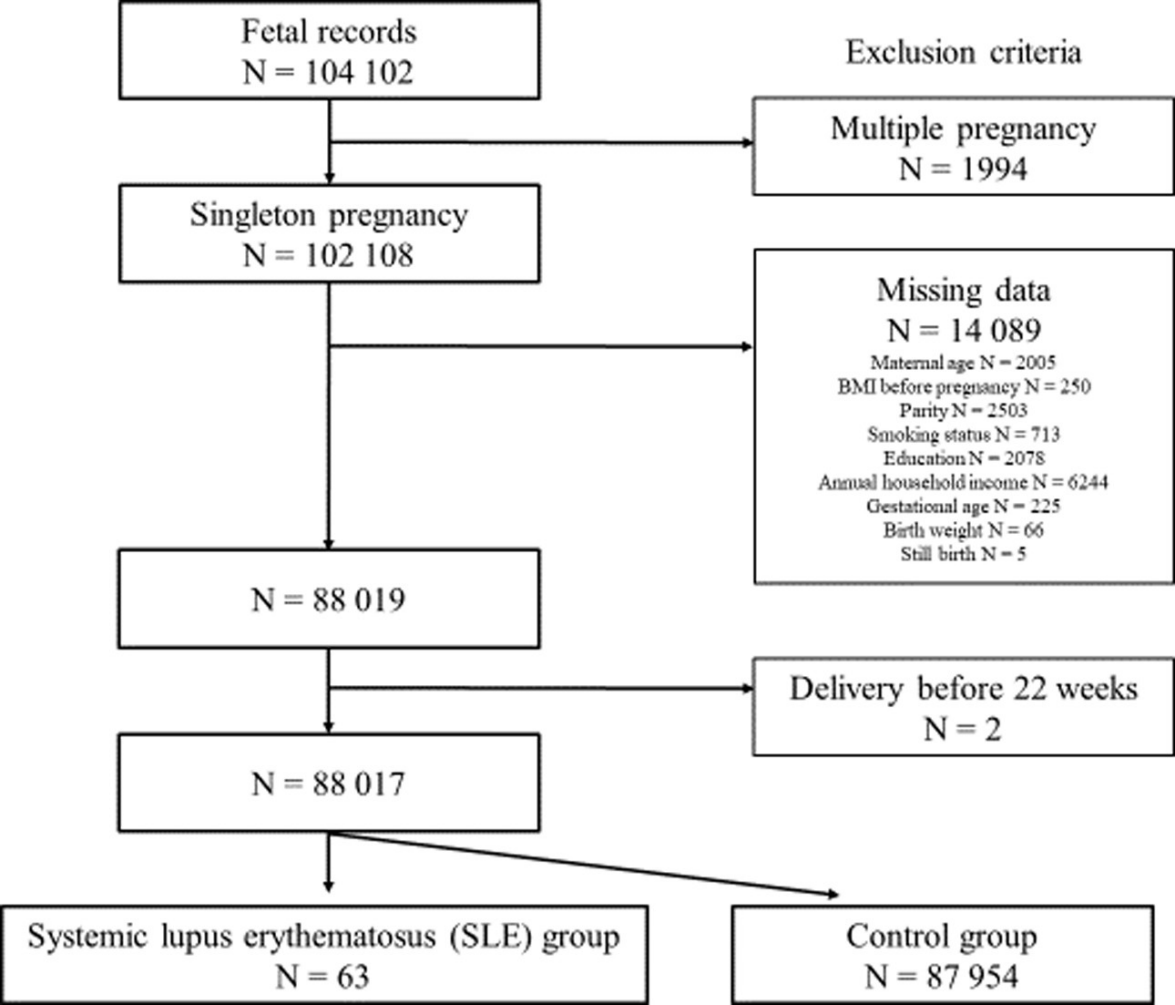
Study enrollment flowchart.

The total number of fetal records of women in the JECS who delivered from 2011 to 2014 was 104,102. After applying the inclusion criteria, 88,017 participants were eligible for the study (Fig 1). Sixty-three participants had SLE, and the remaining 87,954 comprised the general population.

Table 1 summarizes the medical background and obstetric outcomes in the SLE and general population groups. The incidence of PTB before 37 and 34 weeks, LBW < 2500 g and <1500 g, SGA, pPROM, and GH in patients with SLE were 28.6%, 4.8%, 38.1%, 3.2%, 14.3%, 12.7%, and 4.8%, respectively. The incidence of PTB before 37 and 34 weeks, LBW < 2500 g and < 1500 g, SGA, pPROM, and GH in the general population group were 4.6%, 0.9%, 8.0%, 0.5%, 5.1%, 1.1%, and 3.1%, respectively. The prevalence of these outcomes was significantly higher in the SLE group, except for GH.

**Table 1:** Maternal medical background and obstetric outcomes of participants according to systemic lupus erythematosus status.

	Total participants	SLE	General population	p-value
Variable	(n = 88,017)	(n = 63)	(n = 87,954)	
Maternal medical background				
Maternal age (y), mean (SD)	31.3 (4.9)	31.8 (4.4)	31.3 (4.9)	0.456
BMI before pregnancy, mean (SD)	21.2 (3.3)	20.4 (2.6)	21.2 (3.3)	0.035
Nulliparous, % (n)	30.9 (27,169)	50.8 (32)	30.9 (27,137)	0.001
Smoking during pregnancy, % (n)	4.6 (4,074)	1.6 (1)	4.6 (4,073)	0.250
Obstetric outcomes				
Gestational age (weeks), mean (SD)	38.8 (1.6)	37.5 (2.9)	38.8 (1.6)	0.001
Preterm birth (<37 weeks), % (n)	4.6 (4,036)	28.6 (18)	4.6 (4,018)	<0.001
Preterm birth (<34 weeks), % (n)	0.9 (811)	4.8 (3)	0.9 (808)	0.001
Birthweight (g), mean (SD)	3025 (415.7)	2580 (541.2)	3026 (415.4)	<0.001
LBW (<2500 g), % (n)	8.0 (7,032)	38.1 (24)	8.0 (7,008)	<0.001
LBW (<1500 g), % (n)	0.5 (467)	3.2 (2)	0.5 (465)	0.004
SGA, % (n)	5.1 (4,351)	14.3 (9)	5.1 (4,342)	0.001
pPROM, % (n)	1.1 (975)	12.7 (8)	1.1 (967)	<0.001
GH, % (n)	3.1 (2736)	4.8 (3)	3.1 (2,733)	0.449
Still birth, % (n)	0.1 (117)	0 (0)	0.1 (117)	0.772

Abbreviations: SD: standard deviation, BMI: body mass index, PTB: preterm births, LBW: low birthweight infants, SGA: small-for-gestational age infants, pPROM: preterm premature rupture of membranes, GH: gestational hypertension

Table 2 shows that the aORs of PTB before 37 and 34 weeks, LBW < 2500 g and < 1500 g, SGA, and pPROM in the SLE group were 8.1 (95% CI, 4.7–14.1), 5.2 (1.6–16.5), 6.5 (3.9–10.8), 5.4 (1.3–22.4), 2.9 (1.4–5.9), and 12.1 (5.7–25.5), respectively. The aOR of GH was 1.4 (95% CI, 0.4–4.5). The aORs of PTB, LBW, SGA, and pPROM were significantly higher in the SLE group relative to the general population, especially for PTB before 37 weeks, LBW < 2500 g, and pPROM.

**Table 2:** Adjusted odds ratios and 95% confidence intervals of obstetric complications in the systemic lupus erythematosus group.

Obstetric outcomes	PTB		LBW		SGA	pPROM	GH
	<37 weeks	<34 weeks	<2500 g	<1500 g			
SLE group*	8.1 (4.7–14.1)	5.2 (1.6–16.5)	6.5 (3.9–10.8)	5.4 (1.3–22.4)	2.9 (1.4–5.9)	12.1 (5.7–25.5)	1.4 (0.4–4.5)

Abbreviations: aOR: adjusted odds ratios, PTB: preterm births, LBW: low birthweight infants, SGA: small-for-gestational age infants, pPROM: preterm premature rupture of membranes, GH: gestational hypertension

* In the multivariate logistic regression analyses, the general population was used as the reference group, and the results were adjusted for maternal age, BMI before pregnancy, parity, maternal smoking status, maternal educational status, and annual household income.

The results show a higher incidence of PTB, LBW, SGA and pPROM in pregnant women with SLE, compared to those in the general population. Our results, for which we used JECS, a nationwide Japanese prospective birth cohort study, were consistent with those of recent studies [1, 7, 8, 13, 16]. A large Asian nationwide case-control study showed the prevalence of PTB in women with SLE versus a control group was 23.7% vs. 7.6%, OR 3.0 (95% CI, 2.6–3.5) [8]. A large population-based study showed that women with SLE had a 2 to 3 times increase in odds for LBW and SGA [24]. In addition, one of the same studies showed an increased incidence of FGR in women with SLE versus a control group (9.9% vs 4.1%, OR 2.2, 95% CI: 1.9–2.7) [8]. Several previous studies have also shown an increased risk of pPROM in women with SLE [1, 16]. Despite the relatively small number of SLE patients, we found higher odds for adverse obstetric complications than in previous studies. The difference may be due to the comparison with a large general population, thus making the results in the present study more statistically robust and reliable.

Adverse obstetric outcomes such as PTB, LBW, and SGA are thought to be due to placental infarction or the loss of utero–placental blood perfusion. This is because impaired early placental development leads to poor vascularization, resulting in placental ischemia and subsequent endothelial damage [25]. In addition, spontaneous PTB and pPROM are related to either elevated placental corticotropin-releasing hormone, which causes labor through cortisol and prostaglandin [26], or to systemic inflammation from systemic rheumatologic disease, which causes labor as a result of cytokines, prostaglandins, and complement activation [27]. Regarding LBW and SGA, placental ischemia has been related to insufficient fetal growth [28].

Although previous studies have shown increased risk of preeclampsia or eclampsia [8, 16] and hypertensive disorders of pregnancy (HDP) [29], the present study showed no increased risk of GH. This may be accounted for by the difference in criteria to define the complication. We defined GH as elevated maternal blood pressure, but we did not refer to laboratory data; HDP should be defined and characterized considering the assessment of organ function. Thus, the rationale for HDP and eclampsia may differ from that shown as GH in the present study, and careful interpretation of the results is needed. Regarding HDP, further studies are needed that include data regarding organ function. In SLE patients, not only close observation of maternal blood pressure but also close monitoring organ functions during pregnancy may be crucial not only to detect the severity of SLE but also the incidence of HDP, according to previous studies.

The strength of the present study was the calculated aORs for various adverse obstetric outcomes in women with SLE using a large Japanese general population for comparison. This provides more information on the comparative risks faced by pregnant women with SLE. Because of the large study population, including over 80,000 general participants, our results should be reliable. Notably, the aORs of PTB before 34 weeks and of LBW under 1500 g were significantly higher, which suggests that pregnancies in women with SLE may be directly linked to neonatal outcomes. The data suggests the need to appropriately manage pregnancies in women with SLE to improve neonatal outcomes. For example, appropriate evaluation for the risk of PTB, appropriate interventions to prevent PTB and improve outcomes of neonates born before 37 weeks of gestation by vaginal progesterone, antenatal corticosteroids and magnesium sulfate [30–33], and timely interventions to neonates in NICU may lead to better outcomes in neonates born to mothers with SLE.

The present study has several limitations for consideration. First, despite a large sample size from the general population, SLE cases were limited to less than 100, which was not sufficient to evaluate the incidence of still birth or miscarriage. Nevertheless, we could detect the increased risk of PTB, LBW, SGA, and pPROM and we judged the number of SLE patients were enough to the analysis in the present study. Second, variations in SLE treatments and disease activity were not taken into consideration. Differences in treatment or disease activity may affect the occurrence of obstetric outcomes. These factors should be evaluated in further studies in order to detect risk according to stratified groups.

## Conclusions

This study revealed a higher incidence of PTB, LBW, SGA, and pPROM in women with SLE compared to those in a large general population. It is important for care providers to provide the latest data and proper counseling regarding the risks of adverse obstetric complications in women with SLE.
